# Influenza emergence in the face of evolutionary constraints

**DOI:** 10.1098/rspb.2011.1168

**Published:** 2011-07-20

**Authors:** Adam Kucharski, Julia R. Gog

**Affiliations:** Department of Applied Mathematics and Theoretical Physics, University of Cambridge, Cambridge CB3 0WA, UK

**Keywords:** influenza, fitness costs, emergence, transmission, antigenic drift

## Abstract

Different influenza subtypes can evolve at very different rates, but the causes are not well understood. In this paper, we explore whether differences in transmissibility between subtypes can play a role if there are fitness constraints on antigenic evolution. We investigate the problem using a mathematical model that separates the interaction of strains through cross-immunity from the process of emergence for new antigenic variants. Evolutionary constraints are also included with antigenic mutation incurring a fitness cost. We show that the transmissibility of a strain can become disproportionately important in dictating the rate of antigenic drift: strains that spread only slightly more easily can have a much higher rate of emergence. Further, we see that the effect continues when vaccination is considered; a small increase in the rate of transmission can make it much harder to control the frequency at which new strains emerge. Our results not only highlight the importance of considering both transmission and fitness constraints when modelling influenza evolution, but may also help in understanding the differences between the emergence of H1N1 and H3N2 subtypes.

## 1. Background

The two influenza A subtypes common in humans, H1N1 and H3N2, frequently escape population immunity by changing their antigenic properties. Between 1983 and 2009, H3N2 fixed more amino acid changes in antigenic sites than H1N1 [1–4], and vaccines against H3N2 were updated more often than vaccines against H1N1 [5,6]. The higher rate of appearance and fixation of non-synonymous mutations in H3N2 could be owing to a combination of factors, presumably including effective virus population size, and selective pressures according to host prior immunity. However, the root cause of the epidemiological and evolutionary differences between subtypes of influenza in humans remains poorly understood [7,8].

Transmissibility, or more specifically the basic reproductive ratio *R*_0_, the average number of secondary cases generated by the average infective individual in a naïve population, has been much studied for influenza [9,10]. The probability that a strain will become established is determined by its effective reproductive ratio, which depends on *R*_0_ and the level of population immunity. Seasons in which H3N2 was the dominant subtype have been associated with a higher effective reproductive ratio [11,12]; there are also typically more deaths from pneumonia and influenza in seasons where H3N2 is the dominant strain [13]. Transmissibility must be therefore considered as a possible mechanism behind the difference in antigenic drift speeds between the two subtypes.

One strain of influenza may confer partial immunity to another [14], and multi-strain models can be used to study the evolutionary dynamics of the disease [15–17]. Here, we define a new strain to be a virus in which the surface proteins have undergone sufficient change, as a result of mutation, to affect people who were previously immune to it. We will use such a model to examine the role that transmission plays in the emergence of new strains.

While multi-strain models are well established, few allow mutation to directly affect aspects of virus phenotype other than antigenicity. Although partially explored in individual-based simulations [1,18], a previous study [19] introduced a model to investigate the behaviour of influenza evolution when mutation affected the ability of a virus to transmit between hosts.

In studies *in vitro*, single nucleotide mutations in other RNA viruses have resulted, on average, in a fitness reduction [20–22]. If random mutation carries a fitness cost, there is a possibility that mutations associated with antigenic change also affect the phenotype in some other way, reducing viral fitness of antigenic escape mutants through antagonistic pleiotropy [23]. Alternatively, or additionally, ‘deleterious hitchhikers’—mutations elsewhere on the genome that happen to be picked up in the process of emergence of the antigenic escape mutant—could have a negative effect (M. Lässig 2011, personal communication). The ill-fated side branches of influenza's evolutionary tree could be the result of costs incurred by either mechanism (M. Lässig 2011, personal communication; [24]). However, new influenza strains appear every few years [25]; this ongoing antigenic mutation and survival could be possible through subsequent compensatory mutations [26,27].

Gog [19] developed a simple model of these processes to show that a high-level fitness loss could in fact stop viral evolution (‘strain lock’), and that vaccination could also nudge the system into this state. In this paper, a two-tier framework is used to separate the interaction of strains via population immunity, modelled deterministically, and the emergence of new strains, treated as a stochastic process. This improves the realism of the fitness loss and compensatory mutation processes, and makes the model computationally far more tractable, allowing the examination of a variety of assumptions.

We see that although the rate of emergence increases almost linearly with transmission in the absence of fitness constraints, when mutation incurs a cost, small changes in the *R*_0_ of the fully fit virus can have a large impact on emergence. This is an example of phylodynamics [7], the relationship between epidemic and evolution dynamics.

Previous work has shown that often a threshold can exist where vaccination can slow or pause the emergence of new strains [19,28,29] and therefore we also look at how the use of vaccination in slowing antigenic evolution is affected by change in *R*_0_. We show that vaccination can be far more successful in controlling evolution when fitness costs are present, highlighting the importance of considering such constraints when modelling influenza evolution.

## 2. Methods

### (a) A two-tiered model

A first approach to adding fitness costs to a multi-strain model, suggested by Gog [19], was to take the one-dimensional line of strains used in many previous models [16,30] and extend it to a two-dimensional space. Each variant had an intrinsic fitness, as well as an antigenic type (which changed in one direction). Mutation to a new antigenic type caused a reduction in fitness, and compensatory mutations were included so that strains can also regain fitness lost. A simple stochastic approximation was included; a test was imposed on new strains present in less than one individual to ensure that they did not emerge automatically. This meant that stochastic effects were present in a basic way, although the approximation had some drawbacks, which will be discussed here.

Suppose *R*_0_ denotes the basic reproductive ratio of the fully fit virus, i.e. the top level of strains described by Gog [19]. This definition of *R*_0_ will be used throughout this paper. A full fitness strain therefore has an effective reproductive ratio *R* = *R*_0_*S*/*N*, where *S* is the number of individuals susceptible to the strain, and *N* is the population size. Here, as in Gog [19], we assume that antigenic change incurs a fixed fitness cost, so a new strain has an effective reproductive ratio *R*_1_ = *χR* < *R* initially, where *χ* denotes the relative fitness of the mutant, reducing *R* by a factor 0 ≤ *χ* ≤ 1.

Let *I*_0_ be the number of individuals infected with the new strain. If large numbers are infective with the dominant strain, even a low rate of mutation will lead immediately to *I*_0_ > 1. Hence it circumvents the stochastic test, and means a possible ‘overspill’ effect, whereby a circulating strain can prop up its weaker mutant, allowing it to persist long enough to undergo a compensatory mutation, despite the fact that would not necessarily survive if both fitness loss and compensation were modelled as a branching process. Conversely, if *I*_0_ < 1 always, as can happen when the dominant strain is endemic at low numbers, the stochastic test will not allow the weaker mutation to ever emerge if it has *R* < 1. However, it has been shown that if emergence is modelled stochastically, infections with *R* < 1 may still survive long enough to subsequently undergo a compensatory mutation [9]. A further issue, mentioned by Gog [19], is that, for *χ* = 1, there are still two versions of each strain circulating, whereas ideally the two-dimensional space should collapse to a one-dimensional line as *χ* approaches 1, and revert to a simple drift model similar to that of Gog & Grenfell [16].

As a simple model of strain emergence leads to the above issues, yet a fully stochastic process with mutation and compensation is computationally intensive, a balance must be sought. We shall therefore use a two-tiered model [31], focusing on the deterministic and stochastic processes separately.

A discrete-time deterministic model of infection at the population level forms the top tier; this is described in §2*c*. Mutation to a lower fitness strain occurs at a constant rate but the actual emergence of new strains, including compensatory mutation, is dictated by the bottom tier: a stochastic approximation, which has boiled down the details of emergence to a simpler process. This is outlined in §2*b*, and allows calculation of the probability that the new strain appears and causes an epidemic. This probability is then used in the top tier of the model as a test of the viability of a new strain appearing at each point in time.

### (b) Emergence process

**Figure 1.**
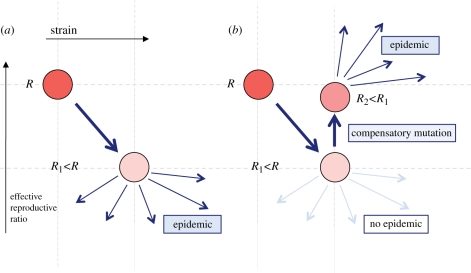
The two routes to emergence: (*a*) despite reduced fitness *R*_1_, virus still causes epidemic; (*b*) no initial epidemic, but compensatory mutation occurs before extinction and virus with fitness *R*_2_ subsequently causes outbreak.

We consider two routes to emergence, as shown in figure 1. In particular, we wish to determine whether a strain with *R* < 1 can mutate before it goes extinct. Similar problems have been tackled for evolution in the face of selection pressure [32] and zoonoses [9,33].

First, we outline a useful result that will be used in the full problem: the probability that a strain with particular fitness fails to cause an epidemic. We assume that *I*_0_ ≪ *S*/*N*, and each infective individual can be considered independent. In the standard susceptible-infective-recovered model, where the effective reproductive ratio *R* is constant, the probability a single infective individual will fail to generate an epidemic is [34]:
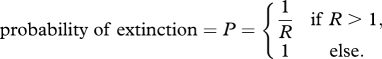


As transmission by each infective individual can be considered independent, we have:2.1
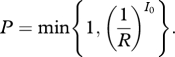


Next, this result can be applied to the full evolutionary process, with compensatory mutation occurring at rate *m*. Let *I*_0_(*t*) be the number of individuals infected with the reduced fitness strain, which has reproductive ratio *R*_1_, and *I*_2_(*t*) be the number of individuals infected with the compensated strain, with reproductive ratio *R*_2_ > *R*_1_. Although this method will work for more general fitness structures, in this paper we assume full fitness recovery, i.e. *R*_2_ = *R*_0_*S*_2_/*N*, where *S*_2_ is the number of individuals susceptible to strain 2.

Following Keeling & Rohani [34], if *I*_0_ = *I*(0) = 1, the probability the reduced fitness strain fails to cause an epidemic, *P*_1_ can be written as a function of the recovery, transmission and compensatory mutation processes. *P*_2_, the probability the full fitness strain fails to cause an epidemic, can be treated the same as *P* above. Rescaling time by the infectious period 1/*γ*, we have:2.2
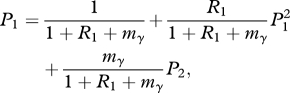
where *m*_*γ*_ = *m*/*γ*. Taking the smallest root in [0,1] of this equation gives us the probability that the reduced fitness strain fails to cause an epidemic [35],2.3
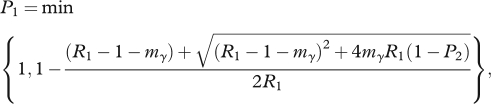
where *P*_2_ = min {1,1/*R*_2_}.

If *I*_0_ ≠ 1 and *I*_0_≪*S*/*N* still, we can again use independence of interactions to obtain:2.4
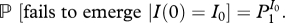


Also note that if *m*_*γ*_ = 0 in equation (2.3), we recover the single strain case *P*_1_ = min{0,1/*R*_1_}, as expected. This is also true if *R*_1_ − 1 − *m*_*γ*_ ≫ 4*m*_*γ*_(1 − *P*_2_)*R*_1_, implying that if there is little or no fitness cost, with *R*_1_ > 1 and *m*_*γ*_ ≪ 1, the probability of emergence behaves as if we had a single strain branching process. Therefore, the step-down and step-up do not have an unnecessary effect if *R*_1_ is not reduced by a fitness cost.

We can use these results to approximate the probability a strain will emerge in our full model. Suppose strain *a* is our frontmost strain (i.e. the one that emerged most recently), at each point in time, we know the prevalence, *I*_*a*_, and susceptibility to a mutant of this strain, *S*_*a*+1_. If mutation occurs at a rate *m*, we would expect to see2.5
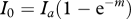
new mutants appear in a single time step. Using the above results, we can therefore calculate *P*_*a*+1_ = *P*_*a*+1_(*R*_1_, *I*_0_), the probability that an infective individual fails to emerge with the new strain *a* + 1, with *R*_1_ = *χS*_*a*+1_/*N*, and *I*_0_ as above.

Computationally, this only requires the following test to be applied at each time step:
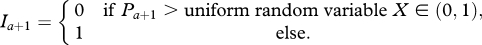


Although we have two routes to emergence (figure 1), and hence two possible values of *R* for *I*_*a*+1_, it is reasonable to assume that if the virus does cause an epidemic, its numbers will end up sufficiently large for it to undergo the compensatory mutation during this epidemic [26,36]. *I*_*a*+1_ therefore represents individuals infective with the full fitness strain.

As in Gog [19], unrealistic strain survival is avoided by setting any strain that decreases in prevalence to *I* < 1, with an *R* < 1, equal to zero. This avoids the decay to infinitesimal levels (i.e. attostrains [37]), and potential re-emergence, which can occur in purely deterministic systems.

### (c) Epidemic process

The second tier of the model keeps track of how immunity changes during epidemics at the population level. To do this, we use a status-based model with one level of fitness only [16], with *S*_*a*_ denoting the number of hosts susceptible to (at least) strain *a*; *I*_*a*_ the number of hosts infectious with strain *a*; and *Λ*_*a*_ the force of infection of strain *a*. We will use a discrete formulation, as it will make it easier to deal with the mutation step at each point in time (which is based on the random number tests in §2*b*). Our system is therefore:2.6
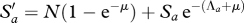
and2.7
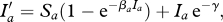
where
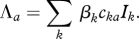


Here *S*′_*a*_ and *I*′_*a*_ represent the values of *S*_*a*_ and *I*_*a*_ at the next time step. Each time step represents one day, and our parameters are scaled as such. We assume a fixed population size *N*, so *μ* acts as both the birth and death rate. *β*_*a*_ represents the transmission rate for strain *a*. Recovery is at a fixed rate *γ*. Cross-immunity is given by the term *c*_*ka*_. We set this to decay exponentially with antigenic distance, and we assume that strains give complete immunity to themselves, so *c*_*ka*_ must be such that *c*_*aa*_ = 1. Table 1 gives the values of the parameters used in the model. The infectious period in §2*b* was given by 1/*γ*, so here *γ* includes both recovery from infection and natural death. However, for typical human influenza parameters, the death rate makes a negligible contribution to *γ*.

**Table 1.** Parameters (sources: *α* [38,39]; *γ* [40]).

variable	description	value
*N*	population size	10 million
*μ*	host birth/death rate	(80 × 365 days)^−1^
*α*	coarseness of antigenic space	0.5
*c*_*ka*_	cross-immunity between strain *k* and *a*	e^−*α*|*k*−*a*|^
1/*γ*	infectious period	5 days
*R*_0_	basic reproductive ratio	varies
*β*	transmission rate	*R*_0_*γ*/*N*
*χ*	relative fitness of mutant	varies
*m*	mutation rate	(4 years)^−1^

A moving strain space, as outlined by Gog [19], is also used to hasten computation. At each point in time all strains with *I* > 0 are identified. By the nature of mutation in the model, these cluster together and hence form a set of adjacent strains on our line. This set is active in the model, in that the above equations only use the variables corresponding to these prevalent strains. We update this active set at each time step. If the backmost (i.e. least recent) strain in the set falls below one infective, then we drop that variable, and if a new strain appears (see §2*b*), we add new variables. There is also a buffer in practice: if a new strain emerges, we create variables associated to the next strain on the line, in anticipation of its emergence.

We approximate the new level of susceptibility by multiplying the immunity with the previous strain by the reduction owing to the imperfection of cross-immunity:
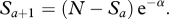


This approximation underestimates immunity (in the form of reduced transmissibility) slightly [19]; this could affect accuracy under a high mutation rate, as more frequent appearance of mutants would mean that the strain space moves faster. However, it is less of an issue for rates similar to those considered in this paper.

Drift speed, used to measure the speed of evolution in our model, is defined as the rate of emergence: the mean number of strains that appear per year, over twenty 50-year simulations. If the system goes extinct, we only look at the behaviour up to the point of extinction. Over many runs, this rate approaches a fixed value for each *R*_0_ and *χ*. We focus on *χ* ∈ [0.6,1], however, as below these values a tendency to lock or go extinct reduces the accuracy of the mean.

## 3. Results

In a manner similar to that of Gog [19], our model exhibits three dynamical behaviours: *locked*, where only one strain circulates; *drifting*, with the continuing emergence of strains; and *extinction*, where no strains remain. Once drift is established, the system is more likely to continue drifting than switch to the other two, especially when *χ* is larger. As *χ* is reduced, a drifting system will have an increasing tendency to fall into a locked state, or go extinct.

First, to check the basic drift dynamics when there is no fitness loss (*χ* = 1), this model is compared with a more basic deterministic model [16] using a continuous version of equations (2.6) and (2.7). In this simpler model, the authors could use an analytical method to calculate drift speed *c*, and it was shown that:
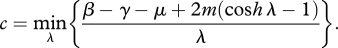


Such a result is not possible for a stochastic system of equations, but using *R*_0_ = *β*/*γ*, we can compare the above theoretical result with the speed observed in our model (using the same values of *γ*, *μ* and *m*). Simulations were run for several values of *R*_0_, with the model deliberately started in a drifting state, and calculated rate of emergence over twenty 50-year runs. This gives a measure of the speed of antigenic drift.

**Figure 2.**
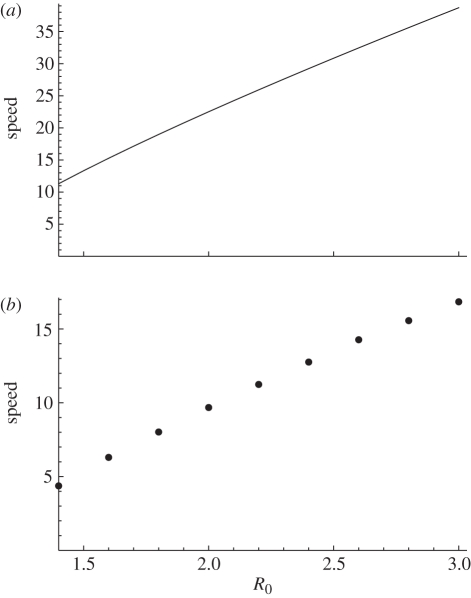
(*a*) Relationship between *R*_0_ and emergence rate predicted by the deterministic model of Gog & Grenfell [16]; (*b*) relationship in our model in the absence of fitness costs.

Figure 2 shows that the qualitative relationship between *R*_0_ and speed of emergence is very similar, with the rate of emergence dependent on *R*_0_ ∈ [1.4,3] in a near-linear way. Although the model presented in this paper evolves at a slower rate, this is most probably owing to the stochastic step that tempers emergence.

**Figure 3.**
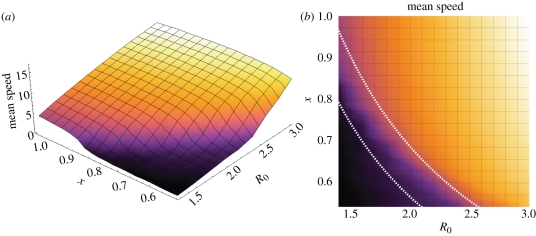
(*a*) Mean strains emerging per year, as a function of *R*_0_ and fitness *χ*. Twenty 50-year runs were used to calculate speeds at 200 points in the *R*_0_/*χ* plane. (*b*) A contour plot of this graph with the theoretical bounds of the drop-off overlaid as dotted white lines. Values of *R*_0_ and *χ* leading to a high rate of emergence are in the top right and to a low rate in the bottom left. The lines are given by the value at which *R*_1_ = *χR*_0_*S*/*N* = 1, for *S* = 0.75 (top) and *S* = 0.9 (bottom), as susceptibility to new strains falls within this range in model simulations.

However, when fitness costs are introduced we see something quite different. The left-hand side of figure 3 shows that when *χ* < 1, the impact of *R*_0_ can become nonlinear. A drop-off appears, along which small changes in transmission can have a disproportionate effect on the speed at which strains appear. Moreover, at the base of this ‘drift cliff’, the relationship is much flatter, and *R*_0_ has even less effect on evolution than it did at when there were no fitness constraints.

It also shows that, if *χ* and *R*_0_ do not vary much between strains, the appearance of a more fit strain with larger *R*_0_ could immediately impact the rate of emergence, as any subsequent antigenic mutant will find it much easier to establish itself.

**Figure 4.**
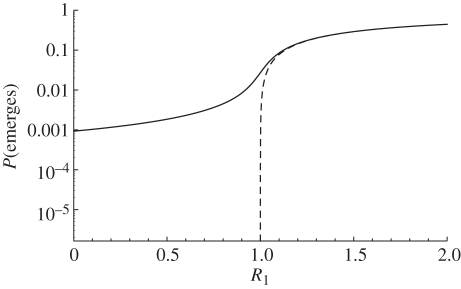
The probability a strain will emerge given *R*_1_ = *χR*_0_*S*/*N*, as calculated from equation (2.3). Here *S*/*N* = 0.9 and *I*_0_ = 1. The dashed line shows the probability that it will emerge in the absence of any compensatory mutations, as in equation (2.1). A strain with *R*_1_ < 1 will find it much harder to cause an epidemic.

We can elucidate this result by considering the role of the stochastic step in the rate of emergence. Figure 4 shows the probability a strain emerges for a particular effective reproductive ratio, *R*_1_ = *χR*_0_*S*/*N*, equal to 1 − *P*_1_ in equation (2.3). As *R*_1_ decreases towards 1, it becomes less likely a new strain will emerge at that step. If *S* were fixed this would imply that, for each *R*_0_, there is a critical value of *χ* below which the appearance of strains is relatively rare. Although a subsequent compensatory mutation and emergence event can still occur, as shown by the solid line in figure 4, this is rare for *R*_1_ < 1 and the long wait for the appearance of a new strain slows down the rate of emergence.

However, in our model, *S* is not fixed because immunity to each strain varies, and depends heavily on other circulating infection. Therefore, we can only estimate this relationship between *χ* and *R*_0_. Simulations show that susceptibility *S* to new strains falls between 0.75 and 0.9, so, by finding the region where *χR*_0_*S*/*N* < 1, this can be used to estimate the location of the drop-off in the rate of emergence. The right-hand side of figure 3 shows that this theoretical result agrees with the simulated behaviour, and hence provides an explanation for the location of the drop-off in emergence.

### (a) Vaccination

Vaccination will be explored by observing how the effects of pulsed vaccination vary with fitness and transmission. For each *χ* and *R*_0_, we let the system settle in a drifting state before vaccinating a fixed proportion of the population chosen at random all at once, at time *T*, with a vaccine most similar to the most recently emerged strain, and cross-reactive with reduced factor e^−*αd*^ for a strain distance *d* away. Hence, if *v* is the vaccine strain, and we vaccinate a proportion *p*,
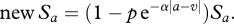


The number of strains that emerge in the interval [*T*,*T* + 365] is then counted and this number is compared with the expected rate of emergence for that *χ* and *R*_0_, as calculated in figure 3, to obtain a relative speed between 0 and 1 as a result of vaccination of the population.

**Figure 5.**
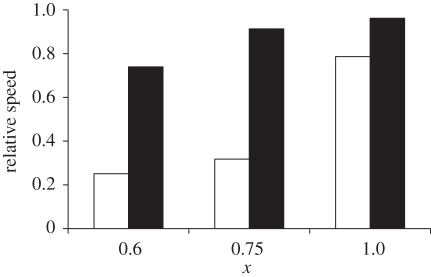
Relative speed of emergence as a result of vaccination of 20% of the population. Calculated as number of new strains in year post-vaccination divided by mean new strains per year if no vaccination. Two hundred simulations were performed for each value, and mean relative speed taken. Open bars, *R*_0_ = 1.6; closed bars, *R*_0_ = 2.

When 20 per cent of the population is vaccinated, the rate of emergence of the strain with *R*_0_ = 1.6 is slowed much more than that of the one with *R*_0_ = 2. Taking the mean from 200 runs, figure 5 shows that while *R*_0_ makes little difference when *χ* = 1, for *χ* < 1, the infection with *R*_0_ = 2 can be far harder to control.

This is because, for a strain with reduced fitness *R*_1_ barely above 1, vaccination can lower susceptibility and put *R*_1_ below 1, and on the slow side of the drop-off. If *R*_0_ = 1.6, *R*_1_ = *χR*_0_*S*/*N* falls into this region. By contrast, with a small increase in transmissibility (in this case *R*_0_ = 2), *R*_1_ is far enough from 1 so as to be far less affected by vaccination. Even a 20 per cent reduction in the number of susceptibles is not enough to push the strain into the area of the drop-off, showing how *R*_0_ has become disproportionately important.

## 4. Conclusions and discussion

We have seen that if the fitness losses that hamper RNA viruses *in vitro* also affect an influenza virus escaping host immunity, drift speed can be affected. Not only does the relative fitness of the virus, *χ*, influence the rate of antigenic escape, there is a feedback between the evolutionary and epidemic processes, with susceptibility and transmission at the population level deciding the impact *χ* has, and vice versa. As seen in figure 3, under fitness constraints, *R*_0_ can become disproportionately important in dictating dynamics. This implies that two strains with identical antigenic space and mutation rates could evolve at very different rates with only a marginal difference in *R*_0_, unlike that required by the deterministic model of Gog & Grenfell [16]. This is illustrated by the drop-off we see between regions of high and low rates of emergence. We have shown that its location can be estimated from the probability that a strain emerges in a branching process; the theoretical prediction coincides well with the simulated results. It also explains the switch between the two stable patterns (locked and drifting) observed by Gog [19]. As *χ* decreases, the probability of switching from a drifting to a locked (or extinct) state becomes much larger, as emergence gets rarer. The drop-off in the overall rate of emergence reflects this reduction in tendency to drift. Further, the existence of such a ‘drift cliff’ between regions of high and low rates of emergence may affect the impact of antivirals, which have been known to reduce the infectious period (and hence the *R*_0_) of influenza [41].

As influenza vaccines are selected around nine months in advance of each hemisphere's annual winter epidemic [42], it would be interesting to see whether there is any indication in historical data that a particularly transmissible season elsewhere in those nine months leads to a higher rate of emergence of new strains, and hence a vaccine update. This may be possible in retrospect, but it would be difficult to monitor the transmissibility of a strain in real-time, with the aim of providing an indication of the level of drift to be expected, as the accuracy of transmissibility estimates are reduced by reporting errors and gaps in surveillance [43]. In addition, calculating *R*_0_ is difficult for seasons with milder epidemics [11]. However, even if the theoretical mechanisms suggested in this paper cannot immediately be applied to real-time prediction, they could be useful in understanding the differences between the emergence of H1N1 and H3N2 strains.

Second, we have seen that the disproportionate impact of transmission continues when the immediate period post-vaccination is considered; under fitness constraints, a small increase in *R*_0_ can make controlling the rate at which new strains emerge much harder (figure 5), as well as controlling the rate at which they spread. However, it also implies that if strains do lose fitness when escaping immunity, vaccination could have a greater benefit than a simpler model would imply.

We have made several assumptions in our model. We have used status-based variables for tractability, simplifying the immune structure of the population. We have also imposed a one-dimensional strain structure on influenza evolution. Although this reflects the sequential appearance of strains implied by the ladder-like phylogenetic tree of influenza A [7,44], the causes of this phylogeny are likely to be complex, with several explanations having been proposed [1,17,18,45] for the observed evolution. By using a simple line of strains, this paper does not address the issue of strain dimensionality. Instead, it assumes that, in the long term, through some mechanism not included here, strain evolution follows a one-dimensional path through antigenic space, with cross-reactivity between strains decaying exponentially as the distance between them increases.

Like several status-based models, we have also assumed that cross-immunity acts to reduce transmission. We do not consider spatial structure, which may also affect how transmission rates impact on strain emergence [12], nor seasonality: this paper presents a single population with influenza constantly circulating. Our parameters, although chosen to be plausible, are also approximate. In particular, mutation rates can affect the steepness of the drop-off: an increase leads to easier emergence and a slightly flatter relationship, whereas a slower rate hinders the appearance of new strains and accentuates the drop (results not shown). Further, the mechanism of fitness loss and regain, via compensatory mutation, is necessarily approximate: a better understanding, ideally quantitative, of how antigenic escape might incur a fitness cost is needed if the effects of evolutionary constraints are to be modelled more accurately. If it was the case that the process of emergence involved several mutations, we would expect that the *R* = 1 boundary is still important in deciding the viability of a new strain. Although simplifications have been made, our framework could easily incorporate new developments in the understanding of influenza or RNA fitness constraints.

Taking a two-tiered approach, we focused on the emergence process and interaction of strains via epidemic dynamics separately, combining a stochastic approach for the former with an ordinary differential equation model for the latter. This allowed us to not only draw attention to the role *R*_0_ can have in dictating a strain's rate of antigenic escape, but also to show that inclusion of realistic evolutionary constraints, as well as careful consideration of the emergence process, can have implications on the use of vaccination in reducing the rate of such escape.
